# Laser Thermochemical High-Contrast Recording on Thin Metal Films

**DOI:** 10.3390/nano11010067

**Published:** 2020-12-30

**Authors:** Elena A. Shakhno, Quang D. Nguyen, Dmitry A. Sinev, Elizaveta V. Matvienko, Roman A. Zakoldaev, Vadim P. Veiko

**Affiliations:** 1 Faculty of Laser Photonics and Optoelectronics, ITMO University, 197101 Saint Petersburg, Russia; elena.shakhno@mail.ru (E.A.S.); kznguyen@itmo.ru (Q.D.N.); evmatvienko@itmo.ru (E.V.M.); zakoldaev@itmo.ru (R.A.Z.); vadim.veiko@mail.ru (V.P.V.); 2 Institute of Automation and Electrometry of the Siberian Branch of the Russian Academy of Sciences IA&E SB RAS, 1 Academician Koptyug ave., 630090 Novosibirsk, Russia

**Keywords:** picosecond laser pulses, direct interference patterning, DOE, thin metal film, Wagner oxidation law, optical contrast

## Abstract

Laser-induced thermochemical recording of nano- and microsized structures on thin films has attracted intense interest over the last few decades due to essential applications in the photonics industry. Nevertheless, the relationship between the laser parameters and the properties of the formed oxide structures, both geometrical and optical, is still implicit. In this work, direct laser interference patterning of the titanium (Ti) film in the oxidative regime was applied to form submicron periodical structures. Depending on the number of laser pulses, the regime of high contrast structures recording was observed with the maximum achievable thickness of the oxide layer. The investigation revealed high transmittance of the formed oxide layers, i.e., the contrast of recorded structures reached up to 90% in the visible range. To analyze the experimental results obtained, a theoretical model was developed based on calculations of the oxide formation dynamics. The model operates on Wagner oxidation law and the corresponding optical properties of the oxide–metal–glass substrate system changing nonlinearly after each pulse. A good agreement of the experimental results with the modeling estimations allowed us to extend the model application to other metals, specifically to those with optically transparent oxides, such as zirconium (Zr), hafnium (Hf), vanadium (V), niobium (Nb), and tantalum (Ta). The performed analysis highlighted the importance of choosing the correct laser parameters due to the complexity and nonlinearity of optical, thermal, and chemical processes in the metal film during its laser-induced oxidation in the air. The developed model allowed selecting the suitable temporal–energetic regimes and predicting the optical characteristics of the structures formed with an accuracy of 10%. The results are promising in terms of their implementation in the photonics industry for the production of optical converters.

## 1. Introduction

Micro and nanotopologies on thin films provide unique possibilities for photonic needs [1] to fabricate diffractive optical elements (DOEs) [2], metalenses [3], nanoantenna arrays [4,5,6,7], etc. The listed applications set requirements for the manufacturing process of each surface element, namely, the shape, size, composition, and optical properties. Typically, electron- or ion-beam lithography techniques are used to process the surface of various materials under well-controlled conditions with the nanoscale resolution [8]. However, these procedures are utterly expensive and also time-consuming, since the processing speeds are quite low.

The direct laser irradiation of metal thin films is a promising approach to meeting the needs. Laser pulses depending on the parameters initiate thermophysical processes such as oxidation, melting, and ablation. However, the diffraction limit restricts the direct laser writing technique. On the other hand, researchers have developed cutting-edge ways to overcome the limit, for instance, direct interference patterning by registration of an interference pattern from two or more laser beams [9,10,11,12,13,14,15,16]. Other most recent experiments tackle this problem by the laser-induced formation of oxide layers on metals of titanium (Ti) [17] and vanadium groups (V, Zr, Ta, et al.) [18,19,20]. The formed oxide layer is optically transparent and carries the following benefits: (i) a single-step way to fabricate a photonic element as a result of oxidation of the film throughout its full thickness (“through” oxidation), (ii) ability to control the process in real time, and (iii) the resolution increase by the reduction of recording laser radiation due to the high-rate formation of a transparent oxide [21]. These advantages are collected in the method called “thermochemical laser recording”, which was developed both for the manufacturing of commercial photonics products [22] and for studying the fundamental aspects of laser-induced thin metal films oxidation to improve the resolution, recording speed, and repeatability [23].

Recently, various technical combinations of the thermochemical laser recording method were realized on Ti films: the direct action of a focused scanning laser beam [24,25], the formation of thermochemical laser-induced periodic surface structures (LIPSSs) [21,23,26,27], or the thermochemical registration of an interference pattern [28,29], including researches by our own group. However, to this day, there is no confirmed theoretical model that could predict the optimal laser processing parameters (e.g., number of laser pulses or fluence) required to fabricate the structure of minimum width while maintaining the maximum contrast. Thus, the results remain semi-empirical and require additional research when changing the recording conditions, e.g., replacement of the laser source, film thickness, or composition.

Hence, this study focuses on the optimization of thermochemical laser recording on thin Ti films supported with theoretical simulation for various metals—Ti, Zr, Hf, V, Nb, and Ta. Periodical structures on Ti films were experimentally recorded by a two-beam interference patterning to verify the simulation result. The proposed model shows the existence of laser regimes with an optimal number of pulses to obtain the minimum structure width with maximum optical contrast.

## 2. Laser Interference Patterning

### 2.1. Experimental Procedure

**Figure 1:**
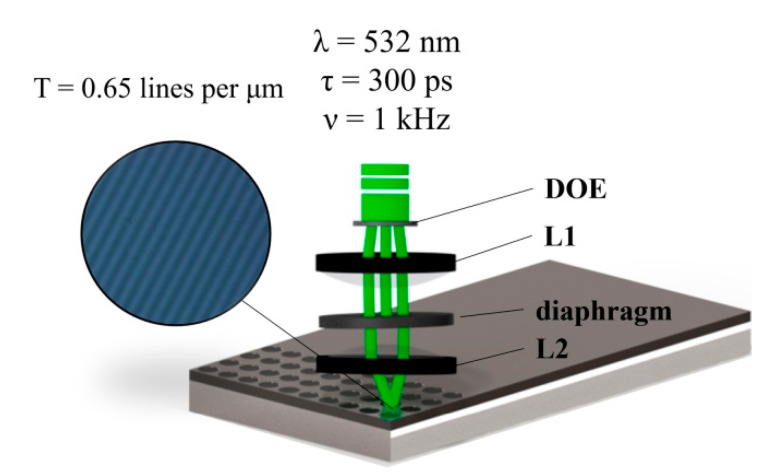
Scheme of the interference laser processing setup, where 300 ps laser pulses provided two-beam interference pattern on Ti film, where a typical pattern with a resolution of 0.65 lines per µm can be obtained. Ti film oxidation was conducted under the irradiation with a fluence of 0.1–0.3 J/cm^2^ with a different number of laser pulses (*N*) from 1 to 10^4^.

Our experiment is an integral part to confirm the simulation results. We used a Ti film with a thickness of 60 nm, which was deposited on a glass substrate by thermal sputtering in a vacuum. A laser interference setup was utilized for the Ti film patterning by a multi-beam interference field (Figure 1). A picosecond laser was used as a laser source. The shape of the beam was Gaussian with the wavelength λ = 532 nm, pulse duration *τ* = 300 ps, maximum pulse energy *E_p_* = 1.3 mJ ± 3%, and repetition rate *ν* = 1 kHz. The laser beam passing through the DOE was divided into several beams with a certain intensity distribution. DOE’s fabrication technique and parameters are given in our previous work [26]. A confocal imaging system consisting of lenses 1 and 2 was applied to realize interference fields in the processing zone. The diaphragm blocked a zero-order maximum, leaving only ± 1 orders to provide two-beam interference in the focal plane of the second lens, *L*_2_. The sample was placed in the focal plane of *L*_2_. The diameter of the formed processing zone was equal to *D* = 300 μm.

We used optical microscopy (Carl Zeiss Axio Imager A1.m, Zeiss, Oberkochen, Germany) to investigate surface structures after interference patterning of Ti film. Atomic force microscope (AFM) (NT-MDT Nanoeducator, NT-MDT SI, Moscow, Russia) was applied to measure the thickness of the oxide layers.

### 2.2. Setting the Simulation Problem

Laser thermochemical recording on thin Ti films requires a rather long (up to ms) exposure to obtain through oxidation due to the feedback complexities of the oxidation process [25,26,27,30,31,32,33,34]. However, in order to avoid the accumulation of heat in the film and its thermal damage, it is preferable to expose the film by several thousand consecutive picosecond pulses with a low repetition rate (around 1000 Hz or lower) [34]. For each sequent pulse, an increase in the thickness of the oxide layer *H* can be determined from the Wagner oxidation law [35]:

(1) $$\frac{\;dH}{dt}=\frac{B}{H}\exp\left(-\frac{T_{a}}{T}\right)\;$$

where *B* is a parabolic rate constant; *T_a_* is an activation energy (in Kelvins); and *T* is an instantaneous temperature value.

To determine the thickness of the oxide layer *H* and its distribution over the film surface, we solved Equation (1) using the “equivalent time” method proposed earlier by Libenson, M.N. [35], which had numerous applications in our and other authors’ works (for example, [24,28,29,30,31,32,33,34]) for calculation of the dynamics of oxidation, taking into account the temperature distribution in the film at the heating *T_heat_* and cooling *T_cool_* stages for each pulse [34]:

(2) $$\left\{ \begin{array}{l} T_{heat}=T_{in}+\frac{q\left(x\right)A\left(x,H\right)\cdot t}{\rho_{me}c_{me}h}\left(\frac{1}{1+\gamma \sqrt{t}}\right)\;\\ T_{cool}=T_{heat}\left(t\right)-T_{heat}\left(t-\tau \right) \end{array}\right.$$

where *ρ_me_*, *c_me_*, *ρ_ox_*, *c_ox_*, *ρ_S_*, and *c_S_* are the densities and heat capacities of the metal layer, oxide layer, and substrate, respectively; *a_S_* is the substrate thermal diffusivity; *h* and *h_me_ =* (*h − H/υ_PB_*) are the thicknesses of the metal layer before and after laser action; *υ_PB_* is the Pilling–Bedworth coefficient, which equals the ratio of the molar volumes of the metal oxide and metal itself, 1.78 for Ti [36]; *T*_in_ is the initial film temperature; *q*(*x*) is the spatial distribution of laser intensity along the transverse coordinate *x*, which was approximated by a sine function; *τ* is the laser pulse duration; *A(x, H)* is the film absorption, which was evaluated for each pulse using the optical matrices method (according to [37]) depending on the oxide layer thickness at the beginning of the pulse action. The heat transfer characteristic (*γ*) from the film to the substrate corresponds to:

(3) $$\gamma =\frac{\sqrt{\pi }}{2}\cdot \frac{\rho_{s}c_{s}\sqrt{a_{s}}}{\rho_{me}c_{me}h_{me}+\rho_{ox}c_{ox}H.}$$

### 2.3. Simulation Procedure

**Figure 2:**
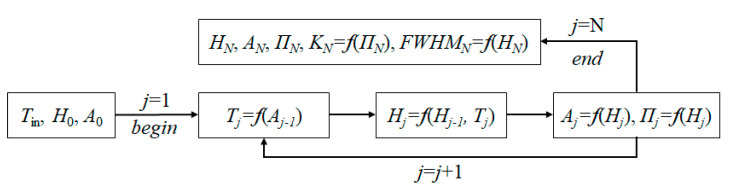
Block diagram of the calculation model. *A*_0_ is an initial value of film absorption depending on an initial film thickness *h*; initial oxide layer thickness *H*_0_ = 0. *T*_j_, *Π*_j_, *K*_j_, FWHM (full width half maximum)_j_ is the film temperature, transmittance, contrast, or element width after the action of the *j*-th laser pulse respectively.

The developed methodology for calculating the results of oxidation after each pulse at the point with the coordinate *x* = *x*_0_ is expressed by the cycle depicted in Figure 2. The calculations were performed in the computer algebra system Mathcad 15. Since the pulse repetition rate under the estimated conditions was quite low [38], the heat accumulation in the film between pulses was not taken into account in this simulation.

The algorithm for calculating the required parameters was built based on Equations (1)–(3) above. The temperature distribution on the metallic film surface during a complete *j*-th pulse (consisting of the heating and cooling phases) as a function of the absorptance can be determined from Equation (2). From that based on the solution of Equation (1) by Libenson [35], one could easily calculate the oxide layer thickness formed by high-temperature oxidation, and the oxide layer thickness is considered as the total thickness of the previously formed oxide layers and the newly formed oxide layer after the *j-*th pulse. Using known parameters about the oxide thickness as well as the remaining metal film below it, and based on Fresnel’s equations, the necessary optical parameters such as the film absorptance and transmittance at the end of the *j*-th pulse can be determined. Once the optical properties and the oxide layer thickness are known, the cycle was reiterated in order to calculate the temperature, the oxide layer thickness, and the optical parameters at the next pulse. Thus, after *N* pulses, the contrast *K* can be calculated as a function of the film transmittance *K* = ((*Π*_max_ − *Π*_min_)/*Π*_max_)⋅100% (where *Π*_max_ is transmission in the center of the irradiated zone and *Π*_min_ is the transmission of the initial film), and FWHM (full width half maximum) can be estimated as a function of the oxide layer thickness distributed along the *x*-axis.

## 3. Results and Discussion

### 3.1. Simulation Results

Since the film temperature is quite sensitive to the small changes in the radiation energy [36], using Equations (1) and (2), we have predetermined the fluence intervals in which the surface temperature in the area of interference maxima does not exceed the melting point of the metal film but is high enough to activate oxidation by oxygen diffusion. For example, for Ti, this temperature range is about 900–2000 K [39], and that corresponds to the fluence in the range of 0.1–0.3 J/cm^2^ for the film thickness of 60 nm. Other parameters used for estimations can be found in Appendix A.

**Figure 3:**
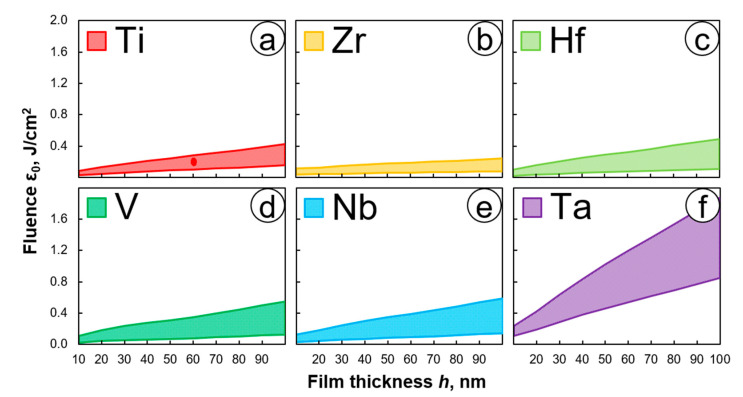
Estimated ranges of laser fluence for different film thicknesses and different materials: (**a**) titanium Ti, (**b**) zirconium Zr, (**c**) hafnium Hf, (**d**) vanadium V, (**e**) niobium Nb, (**f**) tantalum Ta. The red dot at the Ti diagram shows the area of thermochemical recording experimentally defined in [29,34].

Similarly, we found the values of *ε*_0_ applicable for the recording on different metallic films with the same thickness of 60 nm: zirconium (0.1–0.2 J/cm^2^), hafnium (0.07–0.32 J/cm^2^), vanadium (0.08–0.35 J/cm^2^), niobium (0.1–0.4 J/cm^2^), and tantalum (0.5–1.2 J/cm^2^). The estimated ranges of laser fluence for different film thicknesses and different materials (Ti, Zr, Hf, V, Nb, Ta) are shown in Figure 3. The red dot at the Ti diagram shows the area of thermochemical recording experimentally defined in [29,34].

These graphs give us a general view of the ranges of laser fluence necessary to choose the appropriate values when conducting experiments on different metal films. The area for the Ti film is slightly narrower than the others (Figure 3a), and the smallest region was shown for Zr film (Figure 3b). Thus, there will be a few values of fluence that can be selected for a particular thickness of Zr film. The modeling result shows that the films of Hf, V, and Nb possess quite the same range of laser fluence values, although their optical and thermal properties are different (Figure 3c–e). Meanwhile, Ta film has the largest region of the laser fluence (Figure 3f) and thus, there are more possibilities to pick up the right value for the experiment.

### 3.2. Experimental Results

**Figure 4:**
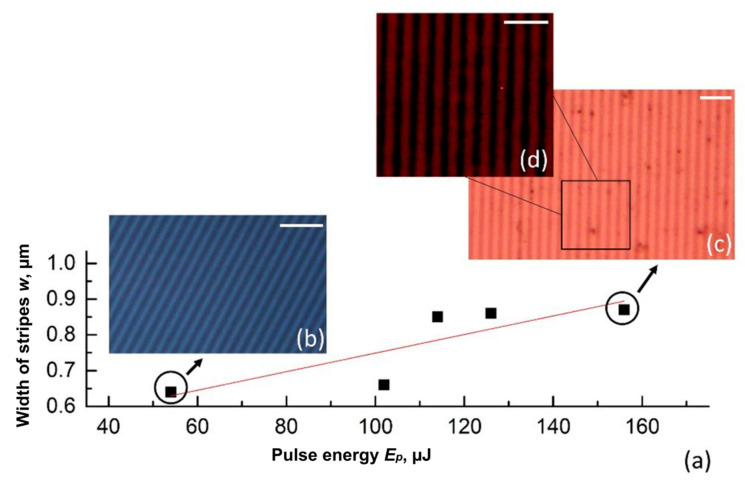
(**a**) The width of stripes (*w*) versus laser pulse energy (*E_p_*). (**b**,**c**) Micro photos of oxidized periodical stripes in reflection light and (**d**) in transmission light. Scale bar is 5 µm.

Intense exposure causes heating of the film material with a given temperature profile, which leads to oxidation near the maxima of the interference distribution. The imprint radius of the interference pattern was ≈150 μm. The Gaussian intensity distribution influenced the uniformity of the contrast of the formed structures, as the structures contrast naturally decreased at the edge of the irradiated region. In this work, we have carried out all studies in the central part of the treatment area limited by the half-width of the treatment zone. Single-pulse action with minimum pulse energy required for the recording (*E_p_* = 54 μJ) was determined as a threshold value (Figure 4). The data obtained allowed us to estimate the range of laser fluence (*ε*_0_ = 4*E_p_*/π*D*^2^) for the oxidation regime 0.1–0.3 J/cm^2^, which is in the agreement with previously published results [34]. Here, we noticed a linear dependence of a single stripe width (*w*) versus pulse energy. The following basic stages of the film modification were observed: (i) at low fluence *ε*_0_ ≤ 0.05 J∙cm^−2^, the film had no visible changes. At moderate fluence range *ε*_0_ = 0.1–0.2 J∙cm^−2^, the contrast bright stripes appeared with *w* of 0.64–0.66 μm (Figure 4b). Then, the fluence increase up to approximately 0.3 J∙cm^−2^ resulted in the broadening of the stripe up to 0.87 μm (Figure 4c). Let us point out that even at maximum available fluence, the formed oxide is not yet transparent, as it can be seen from the transmission microscopy photo (Figure 4d). The following increase of *ε*_0_ over 0.3 J∙cm^−2^ led to the film ablation forming the periodical groves. Therefore, it was impossible to obtain through oxidation for the entire film thickness by single pulse irradiation, and a higher number of laser pulses with a moderate fluence was required.

**Figure 5:**
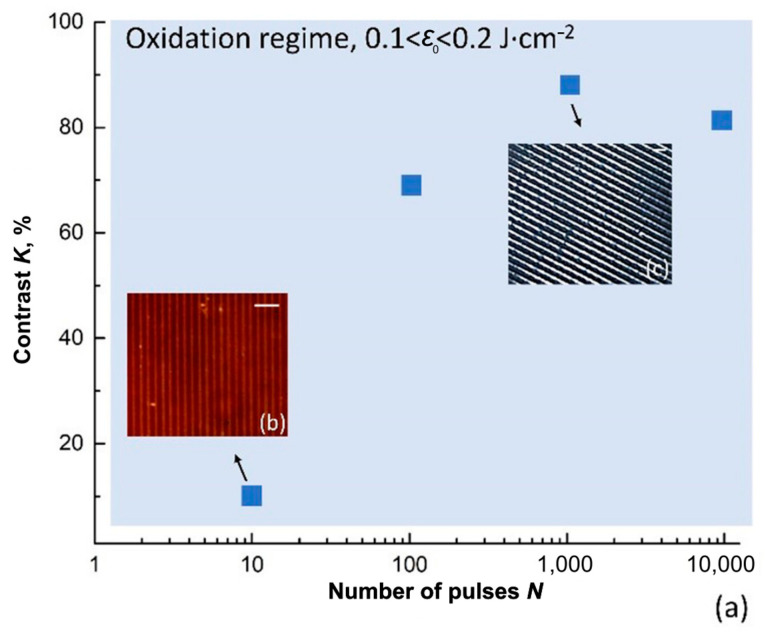
(**a**) The dependence of the optical contrast of fabricated stripes on the number of laser pulses (*N*). The interference pattern was formed in the oxidation regime, 0.1 < *ε*_0_ < 0.2 J∙cm^−2^. The inserted micrographs taken at a constant exposure time (**b**,**c**) shows the formed stripes at 10 and 10^3^ pulses. The scale bar is 5 µm.

Then, we applied the oxidation regime to obtain through by increasing the number of laser pulses (*N*) up to 10^4^. Figure 5 contains data about stripes contrast measured by optical microscopy for different *N*. For non-transparent stripes formed by a single pulse, it was impossible to estimate the contrast by optical microscopy. In the case of *N* = 10, we experimentally observed only slightly transparent stripes with contrast equal to 10% (Figure 5b). At *N* = 100, the formed stripes were characterized by the increased transparency, and the maximum contrast value (≈90%) was obtained at 1000 pulses (Figure 5c). The subsequent increasing of *N* resulted in the contrast decline.

**Figure 6:**
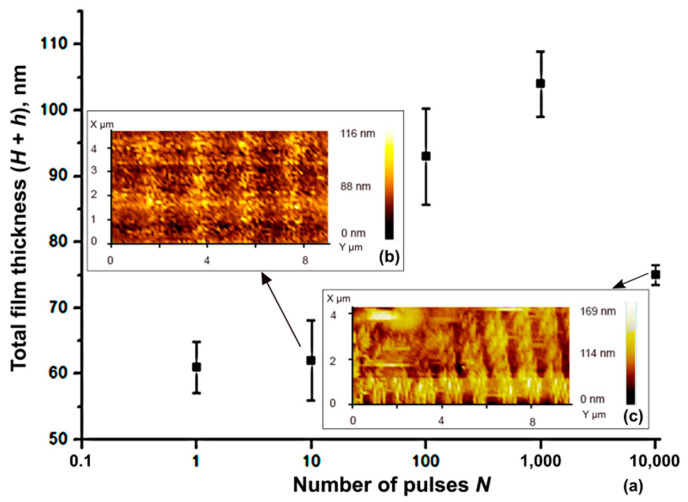
(**a**) Dependence of the total film thickness on the number of pulses *N* from 1 to 10,000. Insets (**b**,**c**) show the atomic force microscope (AFM) images of the structure surface after 10 and 10^4^ pulses, respectively.

In parallel, we evaluated the parameters of the structures based on AFM results, which allowed us to measure the thickness of the formed oxide stripes. Figure 6 presents the growth of film thickness under an exposure of subsequent laser pulses with *N* (1–10^4^). Single-pulse exposure showed only a slight oxide growth, and periodic structures were tracked at 10 pulses (Figure 6b), but these structures were difficult to distinguish with optical microscopy. So, the total film thickness combined from the height of the oxide bump *H* and the thickness of the original film *h* remained approximately 60 nm. After 100 laser pulses, the inhomogeneous and slightly cracked oxide area appears, so the average film thickness reaches up to 90 nm. At 1000 and 10,000 pulses, the structures were homogeneous, but in the region of 10,000 pulses exposure, the thickness lessens (Figure 6c), which is possibly due to thermal degradation or shrinkage of the Ti oxide layer as a result of prolonged laser exposure. The thickness values were 100 and 75 nm, respectively.

Then, AFM thickness measurements were used to calculate the contrast based on the transmission in a two-layer system (TiO_2_-Ti films). The calculations were accomplished in accordance with optical matrices method [37]. The AFM data were used to estimate the actual thicknesses of the formed layers of oxide *H_ox_* and metal *h_me_*:

(4) $$H_{ox}=\frac{H\cdot \upsilon_{PB}}{\left(\upsilon_{PB}-1\right)};h_{me}=\frac{h-H}{(\upsilon_{PB}-1).}$$

The characteristic matrix can be compiled as follows:

(5) $$M(H)=\left[\begin{array}{cc} \cos(\frac{2\pi }{\lambda }n^{\prime }H^{\prime }) & -\frac{i}{n}\sin(\frac{2\pi }{\lambda }n^{\prime }H^{\prime })\\ -in\sin(\frac{2\pi }{\lambda }n^{\prime }H^{\prime }) & \cos(\frac{2\pi }{\lambda }n^{\prime }H^{\prime }) \end{array}\right]$$

where *H*′ is the layer thickness (in our case, it is equal to *H_ox_* or *h_me_*); *n*′ is the complex refractive index of either the oxide or metal, *n_ox_*′ or *n_me_*′, respectively. The characteristic matrix of a multilayer structure can be obtained by sequentially multiplying the matrices of each layer, e.g., specifically for the medium consisting of two thin films as follows:

(6) $$M_{2}=\left[\begin{array}{cc} \cos(\beta_{ox})\cdot \cos(\beta_{me})-\frac{n_{me}^{\prime }}{n_{ox}^{\prime }}\sin(\beta_{ox})\cdot \sin(\beta_{me}) & -i\left(\frac{\sin(\beta_{me})\cdot \cos(\beta_{ox})}{n_{me}^{\prime }}+\frac{\cos(\beta_{me})\cdot \sin(\beta_{ox})}{n_{ox}^{\prime }}\right)\\ -i(n_{me}^{\prime }\sin(\beta_{me})\cdot \cos(\beta_{ox})+n_{ox}^{\prime }\cos(\beta_{me})\cdot \sin(\beta_{ox}) & \cos(\beta_{ox})\cdot \cos(\beta_{me})-\frac{n_{me}^{\prime }}{n_{ox}^{\prime }}\sin(\beta_{ox})\cdot \sin(\beta_{me}) \end{array}\right]$$

where *β_ox_* = (2π/*λ*) *n_ox_*′*H_ox_*; *β_me_* = (2π/*λ*) *n_me_*′*h_me_*. As a result, the optical transmission value of multilayer structure is defined as:

(7) $$\Pi (H)=n_{3}{\left[\frac{2}{M_{2}^{1,1}+n_{3}M_{2}^{2,1}+n_{3}M_{2}^{2,2}}\right]}^{2}$$

where *n*_3_ is the refractive index of the substrate; *M*_2_^(*i*,*j*)^ is the element located in the *i*-th row, *j*-th column of the final characteristic matrix, in our case, a two-layer structure, the layer thicknesses of which were determined by the Formula (4) taking into account the measured value *H*. Finally, the contrast of the recorded structure was defined as the function of the films.

### 3.3. Contrast of Fabricated Structures

**Figure 7:**
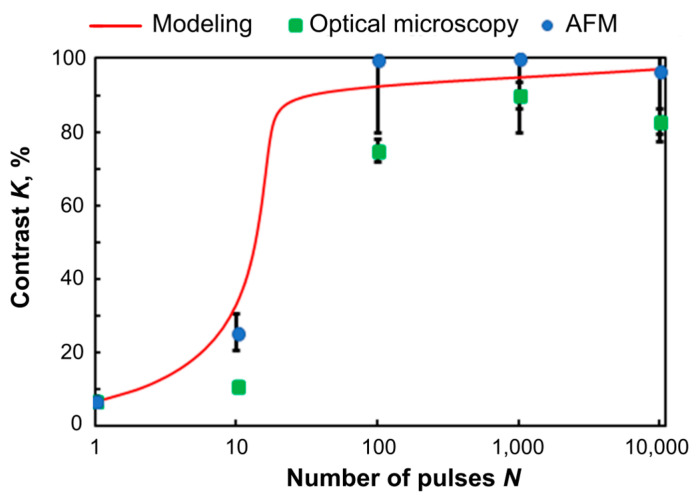
Calculated (**solid line**) and experimentally determined (**points**) dependence of the contrast of the recorded structures on the number of pulses.

We determined that the formed structures are optically transparent, but each applied laser fluence results in a different degree of transparency. The contrast of the recorded thermochemical image is extremely important because it directly determines the efficiency of the optical devices, particularly diffractive optical elements. The obtained results showing the formation of high contrast structures on Ti films under applied experimental conditions were compared between each other and with the simulation results gathered according to the algorithm described above (Section 2.3). Figure 7 presents the fabricated structures’ contrast, which was calculated as the function of the film’s transmittance. The results from all three evaluation methods show strong correlation, e.g., data indicate that the contrast practically does not change after a single laser pulse exposure, but after 100 laser pulses, the contrast sharply increases up to 80%. The maximum contrast of approximately 90% was achieved at 1000 pulses.

The transmittance of light through the thin film is closely related to the amount of TiO_2_ oxide formed during the time being irradiated by the laser beam. The large amount of Ti metal easily reacts to form oxide during the first 30 pulses due to the high level of initial absorption, which is plotted as the fast increasing contrast in Figure 7. At a certain stage, the oxidation becomes more difficult for the small layer of metal between the oxide layer and the glass substrate, since most of the energy from the laser beam is easily transmitted through the film, and the contact with oxygen is mostly hindered by the upper oxide layer itself. Thus, after about 30 pulses, the calculated contrast curve on the graph increases only slightly.

The simulation results (red curve in Figure 7) allowed us to estimate the contrast and compare it to the experimental data. The simulation shows as well that under the selected conditions, the recording contrast reached a value of 90% after exposure to the first 30–100 pulses and changed insignificantly after that, so the effect of subsequent 9900 pulses was excessive.

Figure 7 shows that the combined application of the methods of optical microscopy and AFM makes it possible to assess the contrast of structures, and the analytical model convincingly correlates with the obtained experimental data and can be used to assess the expected value of contrast in other processing modes. Therefore, to obtain the desired highest contrast value of the order of 90%, picosecond laser pulses are sufficient.

## 4. Influence of Various Parameters on the Laser Interference Patterning Performance: The Modeling Results

The correctness of the theoretical model has been proved by the experiments above, from which the optimal number of pulses (or the performance of the laser system) can be found to achieve high contrast with the smallest element size of the thermochemical recorded image. As a result of the calculations, the research can be extended for metallic films with different initial thicknesses (Section 4.4), for other laser sources with different wavelengths (Section 4.5), and for different film materials (Section 4.6). However, before doing that, it is vital to survey the dynamic of the film absorption, which changes nonlinearly during laser oxidation (Section 4.1).

### 4.1. Absorbance

**Figure 8:**
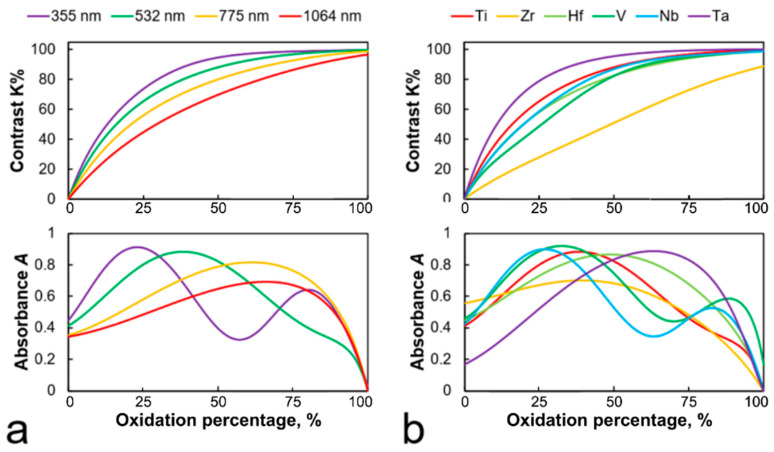
Dependence of the recorded structures contrast and the film absorption on the thickness of the oxidized layer (meaning that oxidation percentage equals to 0% for the initial metal film and to 100% at the through oxidation) (**a**) for different wavelengths of laser radiation for a 60 nm-thick Ti film and (**b**) for different 60 nm-thick films that were oxidized by a radiation with a wavelength λ = 532 nm.

The calculation of the productivity and contrast of the recorded structures have required a predetermination of the optical properties of metal–oxide structures of various thicknesses at different laser radiation wavelengths. Figure 8 shows the dependence of absorbance and contrast of the thermochemical image on the thickness of the oxidized metal layer for radiation wavelengths 355, 532, 775, and 1064 nm (Figure 8a) and different metals (Figure 8b).

The appearance of peaks on the absorption curves *A*(*h_me_*) directly affects the contrast of the recorded structures, as it is shown on Figure 8a that peak absorption values *A*_max_ gradually decline with the wavelength changing from 355 to 1064 nm, which leads to a contrast decrease of recording using an IR light. From here, it can be seen that to obtain a high contrast thermochemical image, it is necessary to use wavelengths from the visible region down to the ultraviolet. Further research will be applied to these regions and described in Section 4.5.

The model shown in Figure 8b also predicts that maximum recording contrast can be obtained on a tantalum film, whereas writing on a zirconium film will result in a lower contrast image compared to other metallic films. Detailed investigation will be presented in Section 4.6.

### 4.2. Number of Pulses

Modeling shows that there exists an optimal number of recording pulses at which the FWHM of a single high-contrast image element is minimal [34]. The existence of such an extremum is determined by thermochemical processes: at a low number of pulses (thus, a thin oxide layer), the film at the center of the irradiated area is oxidized predominantly to the depth than to the edge, and as a result, the FWHM reaches its minimum value at this stage. When increasing the number of pulses, the formation of Ti oxide in the central region also leads to radial heat dissipation [35]. Thus, it increases the temperature at the edge of the irradiated area due to the still high absorption there, which accelerates the oxidation rate and extends the value of FWHM. Our data correlate qualitatively with the results obtained previously by A. Gorbunov’s group on a scanning focused laser beam exposure of Ti films [39].

The following sections present the simulation results for FWHM and the contrast of thermochemical structures under various conditions. The parameters of the laser system used in the simulation correspond to those mentioned above in Section 3.1. Absorption of the film nonlinearly changing during its laser-induced oxidation (see Figure 3) was taken into account in the calculations.

### 4.3. Laser Fluence

**Figure 9:**
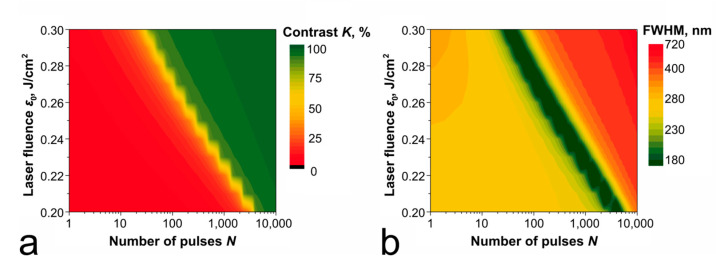
Estimated contrasts (**a**) and respective FWHMs (**b**) of the elements recorded under various conditions on a 60 nm-thick Ti film by a radiation with a wavelength λ = 532 nm.

Significant effect on the contrast image formation and on the optimal number of pulses is exerted by the laser fluence (*ε*_0_): as the simulation results show, varying the fluence within the permissible range of operating values (shown in Figure 3) allows us to reduce the number of pulses required for contrast recording by more than two orders of magnitude (Figure 9), which correlates with experimental results [34]. Modeling was carried out for the regimes ensuring the temperature of the film below the melting threshold (for example, *ε*_0_ ≤ 0.2 J/cm^2^ for a 60 nm thick Ti film) providing high recording accuracy, although oxidation is possible in the liquid phase as well. Our estimations were conducted with the approximation that the fluence level was constant at each pulse. Rough estimations show that the experimentally defined energy deviation of 3% (see the Section 2.1) can yield in the resulting contrast shift of 15–20% during the first 2000 pulses (before the through oxidation), but it will be less substantial subsequently, although further thorough investigation is necessary on this matter.

### 4.4. Initial Film Thickness

**Figure 10:**
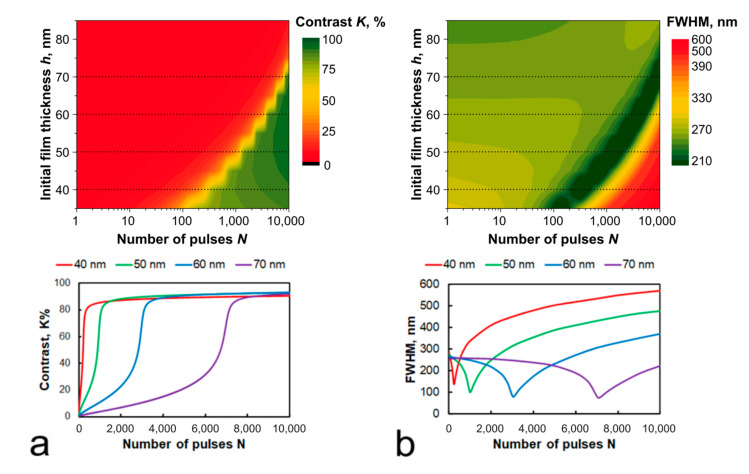
Estimated contrasts (**a**) and respective FWHMs (**b**) of the elements recorded under various conditions on Ti films of various thicknesses by a radiation with the wavelength *λ* = 532 nm and fluence *ε*_0_ = 0.2 J/cm^2^**.**

According to the calculation results shown in Figure 10, the contrast of the thermochemical image reaches its maximum value simultaneously with the formation of elements of the minimum width for Ti films of any initial thickness, and then, it remains almost unchanged under continued laser action. The calculation results indicate the possibility of obtaining high contrast and narrow FWHM values for films of different thicknesses (from 30 to 90 nm). However, achieving high recording quality for thick films requires significantly more pulses (for example, 7000 pulses for a 70 nm thick film, while only 300–500 pulses for a film with an initial thickness of 40 nm). The usage of films with a thickness of less than 30 nm leads to a decrease in contrast.

### 4.5. Laser Wavelength

**Figure 11:**
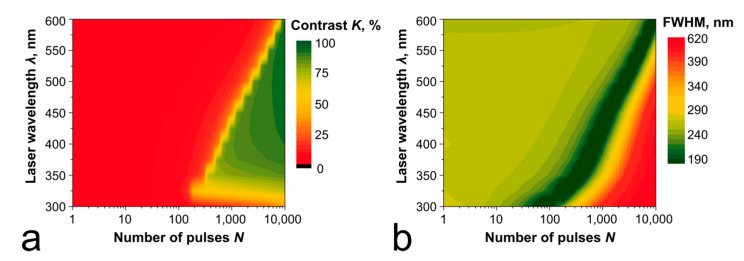
Estimated contrast (**a**) and respective FWHM (**b**) of the elements recorded under various conditions on a 60 nm-thick Ti film by the radiation of different wavelengths with fluence *ε*_0_ = 0.2 J/cm^2^. Data for contrast are shown for a wavelength of the recording laser radiation.

As theoretical estimates show, the action of UV radiation (λ = 355 nm) allows us to relatively quickly (by 400 consecutive pulses) obtain elements with an FWHM of about 70 nm (Figure 11). However, when choosing the wavelength of the recording radiation, it is necessary to acknowledge that absorption of the UV radiation is high not only for the metallic Ti but also for its oxides. A low absorption difference can change the dynamics of through oxidation and limit the contrast of the recorded structures meant for usage as DOEs in the UV bandwidth.

### 4.6. Film Material

To ensure the single-stage laser thermochemical recording, we should use the films of metals that form transparent oxide layers during non-stationary heating: for example, Ti with TiO_2_ [29,34], Zr with ZrO_2_ [40], Nb with Nb_2_O_5_ [41,42], Ta with Ta_2_O_5_ [43,44], V with V_2_O_5_ [45], and Hf with HfO_2_ [46,47].

**Figure 12:**
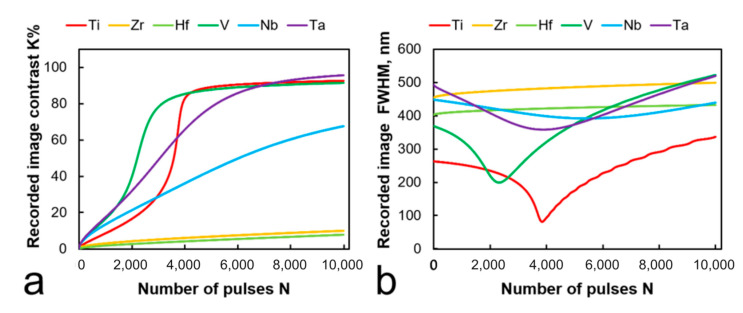
Estimated contrast (**a**) and respective FWHM (**b**) of the elements recorded on the films of various metals. The initial thickness of each film is 60 nm, radiation wavelength is 532 nm, and fluence *ε*_0_ was chosen to match the melting point of each film respectively. Thus, *ε*_0_ equals 0.2 J/cm^2^ for Ti, 0.19 J/cm^2^ for Zr, 0.3 J/cm^2^ for Hf, 0.3 J/cm^2^ for V, 0.4 J/cm^2^ for Nb, and 1.0 J/cm^2^ for Ta.

Figure 12 shows the calculated values of the FWHM and the contrast of the thermochemical image elements depending on the number of pulses for the films of the above-mentioned metals. As expected, among the materials studied, recording on a Ti film seems to be the most efficient: the minimum calculated FWHM is 75 nm when exposed to 3000 pulses (for comparison: FWHM = 430 nm after 1500 pulses on Nb films, FWHM = 360 nm after 3500 pulses for Ta, and FWHM = 200 nm after exposure to 2300 pulses for V). Hf and Zr films only show the steady expansion of the slowly developing thermochemical image, which apparently suggests the necessity of choosing different energy regimes (probably longer pulse durations). Estimations also show that Ti films have the most explicit optimal recording region (meaning recording the narrowest high-contrast elements), but V and Ta films could also provide a high contrast value due to the high initial absorption (see Figure 8).

## 5. Conclusions

The developed and experimentally verified analytical model allowed us to estimate the optimal range of parameters for the efficient recording of high-contrast submicron elements by laser interference thermochemical recording on a variety of metal films with transparent oxides.

The fluences of picosecond pulses required for laser interference thermochemical recording vary from 0.1 to 2 J/cm^2^ depending on the material, the initial film thickness, and the recording radiation wavelength. The developed model predicts the resolution and contrast characteristics of the formed image, and it suggests the parameters with an accuracy of 10%, which is confirmed by the results of AFM and optical microscopy of the actual experimental samples.

The simulation results indicate that for the films of any initial thickness, there is an optimal number of successive pulses, at which the sizes and contrast of the recorded structures are in a perfect balance. To obtain the smallest element size, it is advisable to use films that are as thin as possible, although to achieve high contrast, it is necessary to use films with a thickness not less than 30 nm, as a further decrease in the film thickness leads to the contrast plummeting. Thus, recording on thin (with an initial transmission of about 5–10%) films of metals with low thermal diffusivity (Ti and V) by radiation in a spectral range from UV to green seems to be a priority. The direct interference patterning in these regimes is expected to result in the formation of periodic structures with a minimum size of a single element of down to 70 nm, a contrast of up to 90–95% in the visible range, by 200–400 ms of sequential exposure to picosecond pulses.

## Data Availability

The data presented in this study are available on request from the corresponding author.

## Appendix A

**Table A1:** Physical and chemical properties of metals (and their oxides) from the IV and V groups of the periodic table of elements.

Properties	Symbol	Units	References	Ti	TiO_2_	Zr	ZrO_2_	Hf	HfO_2_	V	V_2_O_5_	Nb	Nb_2_O_5_	Ta	Ta_2_O_5_
Density	*ρ*	kg/m^3^	[48]	4506	4260	6520	5680	13,300	9680	6000	3360	8570	4600	16,400	8240
Heat capacity	*c*	J/(kg∙K)	[48]	523	712	278	480	144	120	489	276.5	265	600	140	306
Thermal conductivity	*κ*	W/(m∙K)	[48]	21	10	22.7	2.2	23	1.1	30.7	1.5	53.7	1	57.5	0.4
Thermal diffusivity (×10^−^)	*a =* *κ/(ρc)*	m^2^/s		0.89	0.33	1.25	0.08	1.20	0.09	1.05	0.16	2.36	0.04	2.50	0.02
Melting temperature	*T_m_*	K	[48]	1941	2116	2128	2988	2506	3031	2183	963	2750	1785	3290	2145
Molar mass	*M*	g/mol	[48]	48	80	91	123	178.5	210.5	51	182	93	256.8	181	442
Pilling-Bedworth ratio	*υ_PB_*		[36]	1.76		1.57		1.62		3.25		2.74		1.171	
Parabolic constant (×10^−7^)	*B*	m^2^/s	[35,46,49,50,51,52]	330,000		0.0175		0.00535		15.6		0.159		0.0102	
Activation temperature	*T_a_*	K	[35,46,49,50,51,52]	33,000		12,890		18,117.8		19,124.4		13,346.2		12,761.6	
Refractive index (at 532 nm)	*n*		[53]	2.48	2.45	2.33	2.17	2.48	2.12	3.92	1.88	2.22	2.37	1.14	2.16
Extinction coefficient (at 532 nm)	*k*		[53]	3.35	0	1.5	0	3.04	0	3.13	0.05	3.12	0	4.72	0
